# Case of Recovery after Ruptured Uterus with Protrusion of Intestine

**Published:** 1873-10

**Authors:** 


### Case of Recovery after Ruptured Uterus, with Protusion of Intestine.

A case of the above occurrence recently formed the subject of a paper read before the Reading Pathological Society by Mr. O. Lowsley, of that place. The following may be taken as affording a brief epitome of the facts of this somewhat remarkable case :—Mr. Lowsley was called on November 11th last to a patient in her fifth labor. About fifteen minutes after the birth of a male child, as the placenta did not come away, he introduced his hand to the insertion of the cord, and made gentle traction on it, but it gave way from being very gelatinous. Feeling something in contact with his hand, resembling a portion of the placenta, he brought it down, and found, to his suprise, that it was in reality a loop of intestine, which he immediately returned. The patient soon afterwards began to exhibit symptoms of collapse. Some brandy and opium were administered. A consultation was held with a neighboring practitioner, Mr. Moxhay, and the placenta was removed. The symptoms of collapse became much more marked, and the worst prognosis was formed. The patient, however, recovered from her collapsed condition, and ultimately (December 20th) was reported as quite well. It appeared that she had been suffering for some time before her confinement from uterine discharges, attended with some bearing-down pain, and on one occasion before the child was born, she felt much pain, and thought that something had given way, and this was followed by symptoms of collapse for some hours. Mr. Lowsley was quite positive that immediately on passing the hand into the uterus he encountered the intestine, and he was equally clear that it did not pass through any opening in the wall of the vagina.—*London Lancet.*

